# Child and adolescent psychiatry patients coming of age: a retrospective longitudinal study of inpatient treatment in Tyrol

**DOI:** 10.1186/s12888-016-0910-x

**Published:** 2016-07-08

**Authors:** Martin Fuchs, Georg Kemmler, Hans Steiner, Josef Marksteiner, Christian Haring, Carl Miller, Armand Hausmann, Kathrin Sevecke

**Affiliations:** Department of Child and Adolescent Psychiatry, Center of Psychiatry and Psychotherapy, Medical University of Innsbruck, Christoph Probst Platz, Innrain 52, 6020 Innsbruck, Austria; Department of General and Social Psychiatry, Center of Psychiatry and Psychotherapy, Medical University of Innsbruck, Innrain 52, 6020 Innsbruck, Austria; Department of Psychiatry and Behavioral Sciences, Stanford University School of Medicine, Stanford, CA 94305-5101 USA; Department of Psychiatry, LKH Hall, Milser Str. 10, 6060 Hall in Tirol, Austria; Department of Psychiatry, Bezirkskrankenhaus Kufstein, Endach 27, 6330 Kufstein, Austria

**Keywords:** Mental health, Epidemiology, Longitudinal course, Childhood, Adolescence, Diagnostic shift

## Abstract

**Background:**

Mental illness is a common phenomenon at all ages. Various independent studies have shown that psychopathology is often expressed on a continuum from youth to adulthood. The aim of our study was to demonstrate a) the frequency of admission of former child and adolescent psychiatry inpatients (CAP-IP) to adult inpatient mental health facilities, and b) a potential longitudinal diagnostic shift. This is the first Austrian study designed to shed light on these issues.

**Methods:**

Nearly 1000 inpatient cases at a specialized child and adolescent care center were analyzed. These cases were then tracked using data matching with registry data from adult psychiatric institutions. Overall, our observational period was 23 years.

**Results:**

26 % of our sample of former CAP-IP used psychiatric inpatient mental health services as adults, thus indicating chronicity or reoccurrence. In line with previous literature, there were patients who stayed in the same diagnostic category as well as patients with a diagnostic shift from childhood to adulthood.

**Conclusions:**

Childhood and adolescence is a very important period for early intervention and prevention of mental illness. Our findings support the notion of the continuity of psychopathology from youth into adulthood.

## Background

Numerous epidemiological studies have investigated the prevalence of mental illness in childhood and adolescence. Recent results have consistently shown that mental disorders are relatively common phenomena among children and adolescents, affecting at least 15 % of the youth population in terms of point prevalence. The lifetime prevalence of mental disorders from childhood until the age of majority is somewhat higher, affecting on average 25 to 30 % of young people at least once [1–6].

A psychiatric disorder in childhood or adolescence has direct effects on the development and acquisition of skills relevant for coping and self-protection; it also substantially increases the likelihood in adulthood of experiencing a variety of health-related, economic and social disadvantages [7–13]. More than half of all adult patients with mental illness retrospectively report that first symptoms occurred during childhood and adolescence and that there was a chronic progression into adulthood [14, 15].

Population-based epidemiological surveys describe two basic patterns of diagnostic development from childhood to adulthood. Some patients stay in the same diagnostic category when coming of age, the other group of patients experiences a diagnostic change in such a way that certain juvenile mental disorders are precursors of different disorders in adulthood. Patients with dissocial, depressive, anxiety, hyperkinetic, substance-related and schizophrenia spectrum disorders mostly stay in the same diagnostic class, while affective or anxiety disorders during adulthood seem to follow externalizing disorders in childhood and adolescence [9, 16–23]. While these findings were gathered in large populational samples, relatively few studies have systematically examined the diagnostic progression of former CAP-patients being referred to adult mental health facilities [24]. The only European large-scale study found was conducted in Italy and found around one fifth of former CAP-patients to be treated in adult mental health institutions [25].

Studies on the development of psychiatric disorders in the community have shown that mental disorders in adulthood are found in approximately one third of all former child and adolescent psychiatric patients [26, 27].

To our knowledge, no study to date has investigated the course of psychiatric disorders in Austrian children and adolescents. The goal of this study was thus to fill this gap and gain insights into the longitudinal development and readmission rates of a cohort of former child and adolescent psychiatric patients. The findings would then be compared with other data in Europe and be used to assess the care we currently provide patients.

## Methods

### Study population and inclusion criteria

The Department of Child and Adolescent Psychiatry is a specialized facility with a public service mandate for the Austrian federal state of Tyrol. It is the only hospital in the state with inpatient services for minors with mental health problems. In 2012, Tyrol’s population was 715,888, with 18.3 % (131,156) under the age of 18 (source: tirol.gv.at). Our sample included all patients who received a psychiatric diagnosis and were treated as inpatients at least once at the Department of Child and Adolescent Psychiatry in Innsbruck between 1989 and 2007. The sample is thus representative of the mental health inpatient treatment of minors in Tyrol. Electronic patient records were evaluated, and the primary psychiatric diagnosis was extracted. At the point of first diagnosis (data point one), all patients were minors, i.e. under the age of 18.

### Diagnostic measures

Psychiatric diagnoses were based on ICD-10 Chapter V. Some patients’ primary diagnoses were coded according to ICD-9 and were converted to ICD-10 using mapping tables. Inpatient cases prior to 1989 could not be included because electronic documentation had not yet been implemented. When a patient had more than one admission to the inpatient unit of the Department of Child and Adolescent Psychiatry, the main diagnosis of the first admission was used. If a patient had more than one admission as an adult, the diagnosis of the last admission was used for the study. For some calculations, we grouped the diagnoses into the broader categories “internalizing disorders” (affective disorders and anxiety disorders) and “externalizing disorders” (hyperkinetic disorders and conduct disorders), which is a common practice in child and adolescent psychiatry [2].

### Study design

The study was designed to assess former CAP-IP retrospectively, longitudinally and cross-sectionally, including follow-up treatment. We investigated whether the children and adolescents in our representative sample of minors with a mental health inpatient diagnosis (data point one) received a psychiatric inpatient diagnosis again as adults (data point two). Additionally, diagnostic transitions between data points one and two were analyzed.

Via data matching using reference dates in 2009 and 2012, we determined whether the patients in our sample had been treated again as inpatients at one of the psychiatric facilities in Tyrol (data point two). Data from all inpatient services in the state were included.

The primary psychiatric diagnosis in adulthood was compared with the diagnosis from childhood or adolescence. In order to be included in the study, patients had to be 18 years of age or older at the date of assessment (data point two); at the second reference date, patients were thus at least 22 years old. In total, diagnoses covering a period of 23 years (1989 to 2013) were tracked. For data matching, social security numbers, names, and dates of birth were used. This permitted the tracking of individuals whose names had changed, e.g. due to marriage.

### Statistical methods

All statistical analyses were performed with SPSS, version 21. The chi-square test was used to test for an association between the diagnostic group at first admission (to the child and adolescent psychiatry facility) and readmission (yes/no) in adulthood. The same test was also applied to investigate associations between diagnostic group in childhood or adolescence (A) and diagnostic group in adulthood (B). Specifically, the following hypotheses were tested:

1. Is there any association between diagnosis A and B? (Null hypothesis: A and B are unrelated.)
2. Does the proportion of patients whose diagnosis remained stable from childhood/adolescence to adulthood (A = B) depend on the diagnosis in childhood/adolescence?

In addition, a logistic regression analysis was performed to test the combined effect of socio-demographic and clinical variables in childhood/adolescence on readmissions in adulthood (yes/no). Significant predictors were selected by means of the stepwise backward elimination method. Odds ratios (OR) were used as a measure of effect size. All statistical tests were performed at a 0.05 level of significance.

## Results

### Sample

A total of 1046 children and adolescents were admitted as inpatients to the Department of Child and Adolescent Psychiatry, Medical University of Innsbruck, at least once between 1 January 1989 and 30 June 2007. Of these, 50 adolescents were excluded because they had already reached the age of 18 years at first admission. A further nine patients had died before their 18^th^ birthday. Thus, a total of 987 patients were eligible for statistical analysis. Demographic and clinical data for these patients are shown in Table 1. Mean age at first admission was 13.8 years (SD 3.2 years), ranging from 2.83 to 17.98 years. There was an almost equal proportion of male (48.5 %) and female (51.5 %) patients.

**Table 1:** Patients admitted to child and adolescent psychiatry unit: patient characteristics and diagnoses

Variable	Category	*N*	%
Age (years)	≤10	204	20.7
	11–14	335	33.9
	15–17	448	45.4
Gender	Male	479	48.5
	Female	508	51.5
Number of admissions	1	424	43.0
	2–9	457	46.3
	10 or more	106	10.7
Cumulative duration of	≤8 weeks	645	65.3
inpatient stays	>8 weeks	342	34.7

**Fig. 1:**
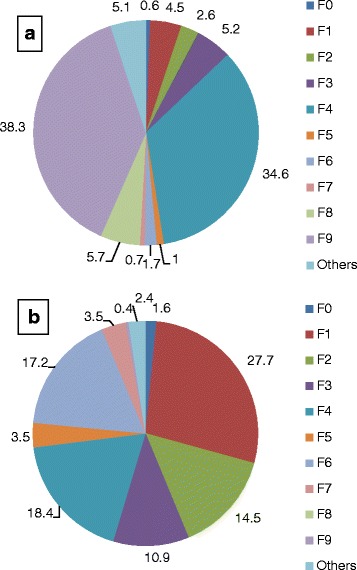
Distribution of diagnostic groups^a^ at first admission to CAP unit and at readmission as adults. **a** CAP inpatients (*n* = 987). **b** Former CAP inpatients readmitted as adults (*n* = 256). ^a^ F0: Organic, including symptomatic, mental disorders; F1: Mental and behavioral disorders due to psychoactive substance use; F2: Schizophrenia, schizotypal and delusional disorders; F3: Mood and affective disorders; F4: Neurotic, stress-related and somatoform disorders; F5: Behavioral syndromes associated with physiological disturbances and physical factors; F6: Disorders of adult personality and behavior; F7: Mental retardation; F8: Disorders of psychological development; F9: Behavioral and emotional disorders with onset usually occurring in childhood and adolescence; Numbers shown are percentages

Almost 40 % of the 987 patients presented with an ICD-10 diagnosis of group F9 (psychiatric disorders with onset in childhood or adolescence), and about one third showed an F4 diagnosis (neurotic, stress-related and somatoform disorders). Approximately 5 % of the admissions fell into each of the diagnostic categories F1 (psychoactive substance abuse), F3 (affective disorders) and F8 (developmental disorders), respectively. All other diagnoses occurred rather infrequently (Fig. 1). When the diagnoses were grouped into internalizing disorders, externalizing disorders and “others”, 61.2 % fell into the first category, 15.5 % into the second, and 22.3 % were classified as “others”.

There were significant gender differences between the diagnostic groups (*χ*^2^ = 83.8, d.f. = 10, *p* < 0.001). In particular, 64.2 % of patients with an F4 diagnosis (neurotic, stress-related and somatoform disorders) were female, whereas over 60 % of patients with an F9 diagnosis (psychiatric disorders with onset in childhood or adolescence) were male (60.6 %). Even larger gender differences were seen when CAP-IP diagnoses were divided into internalizing disorders and externalizing disorders: 59.4 % of patients with internalizing disorders were female, and 77.1 % of patients with externalizing disorders were male (Chi^2^ = 65.4, *p* < 0.001).

### Readmissions in adulthood

A total of 256 patients (26 % of 987) had at least one readmission to a psychiatric treatment unit as adults. The number of adult readmissions per person ranged from 0 to 52, with a mean of 0.52 readmissions. The mean age of the former CAP-IP at the reference date (31 December 2012) was 29.4 years, so the average observation time in adulthood was 11.4 years (SD 5.0 years). The mean number of readmissions per person and year amounted to 0.52/11.4 = 0.045 or 4.5 % (95 % confidence interval 2.5–6.5 %).

An overview of adult readmissions in relation to diagnostic group as CAP-IP is given in Table 2. Overall, there were no significant differences in readmission rates between diagnostic groups (*χ*^2^ = 9.95, d.f. = 10, *p* = 0.445). However, when comparing the individual diagnostic groups with the largest diagnostic group, F9 (psychiatric disorders with onset in childhood or adolescence), a significantly higher readmission rate was observed for patients with a CAD of F1 (psychoactive substance abuse) compared to F9 (38.6 % vs. 24.1 %, respectively, OR = 1.98, *χ*^2^ = 4.39, *p* = 0.036). No other diagnostic group differed significantly from group F9. When divided into internalizing, externalizing and other disorders, readmission rates of patients with externalizing disorders (19.6 %) were found to be slightly lower than those of patients with internalizing (27.2 %) or other disorders (27.0 %), but without reaching statistical significance (Chi^2^ = 3.78, *p* = 0.151).

**Table 2:** Relationship between diagnosis in childhood/adolescence and readmissions as adults

Diagnostic group in childhood and/or adolescence^a^	Total number of patients	Readmission as adults	
		*N*	%
F0:	6	2	33.3
F1:	44	17	38.6
F2	26	9	34.6
F3	51	17	33.3
F4	341	87	25.5
F5	10	1	10.0
F6	17	6	35.3
F7	7	1	14.3
F8	56	13	23.2
F9	378	91	24.1
Others (no F diagnosis)	50	12	24.0
Total	986	256	26.0

^a^F0: Organic, including symptomatic, mental disorders; F1: Mental and behavioral disorders due to psychoactive substance use; F2: Schizophrenia, schizotypal and delusional disorders; F3: Mood and affective disorders; F4: Neurotic, stress-related and somatoform disorders; F5: Behavioral syndromes associated with physiological disturbances and physical factors; F6: Disorders of adult personality and behavior; F7: Mental retardation; F8: Disorders of psychological development; F9: Behavioral and emotional disorders with onset usually occurring in childhood and adolescence

The joint effect of socio-demographic and clinical variables in childhood/adolescence on readmission in adulthood was investigated by multiple logistic regression. Findings are shown in Table 3. Significant predictors of readmission were age at last admission to the University Hospital of Child and Adolescent Psychiatry (more than threefold higher odds for those admitted or readmitted to CAP at age ≥ 13) and total number of admissions (twice higher odds for patients with ten or more admissions in childhood or adolescence compared to those with only one admission), whereas neither gender nor cumulative duration of inpatient stays showed a significant effect. Similarly, the particular diagnostic group – both in terms of the usual classification from F0 to F9 and when grouped into internalizing, externalizing and other disorders – showed no significant effect. Patients with externalizing disorders showed slightly but not significantly lower readmission rates than those with internalizing disorders (OR = 0.677, *p* = 0.098).

**Table 3:** Effect of socio-demographic and clinical variables in childhood/adolescence on readmission in adulthood: results of multiple logistic regression^a^

	Odds ratio	95 % confidence interval	Wald	df	*p*-value
Age at last admission to CAP			29.87	1	<0.001
<13 years (reference group)	1.000				
≥13 years	3.661	2.299–5.830	29.87	1	<0.001
Gender			1.64	1	0.201
Male (reference group)	1.000				
Female	0.817	0.599–1.114	1.64	1	0.201
Number of admissions to CAP			11.77	2	0.003
One (reference group)	1.000				
2–9	1.380	1.003–1.897	3.91	1	0.048
≥10	2.219	1.392–3.537	11.55	1	0.001
Diagnostic group			3.44	2	0.179
Internalizing disorders	1.000				
Externalizing disorders	0.677	0.426–1.075		1	0.098
Others	1.085	0.761–1.547		1	0.378

*CAP* Child and Adolescent Psychiatry

^a^variables not included in the model: age at first admission to CAP (Wald = 2.53, d.f. = 1, *p* = 0.112) and cumulative duration of hospitalizations (Wald = 0.49, d.f. = 2, *p* = 0.781)

### Diagnosis in adulthood

For patients readmitted to a psychiatric hospital as adults, an overview of the diagnoses in adulthood is given in Fig. 1. The largest diagnostic group among readmitted patients was F1 (substance abuse), followed by F4 (neurotic, stress-related and somatoform disorders) and F6 (personality disorders). More details are given in Fig. 1.

### Diagnostic transitions

A question of particular interest was the relationship between the diagnostic category at the time of admission to the Department of Child and Adolescent Psychiatry and the diagnosis at the time of readmission as an adult.

A summary of the findings is given in Table 4. Diagonal entries denote subjects with stable diagnosis (e.g. with an F1 diagnosis both in CAP and in adulthood), whereas off-diagonal entries indicate subjects who showed a change in diagnosis, e.g. from F1 diagnosis in childhood/adolescence to F2 diagnosis in adulthood. Frequently occurring diagnostic transitions are highlighted.>

**Table 4:**
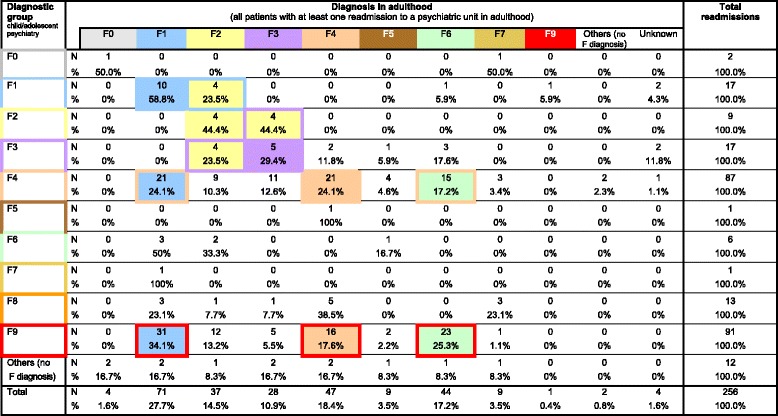
Transition from diagnosis in childhood and adolescence to adult diagnosis^a,b^

^a^Transitions occurring most frequently are highlighted (F4, F9: all transitions with *n* ≥ 15, for all other diagnostic groups: *n* ≥ 4

^b^F0: Organic, including symptomatic, mental disorders; F1: Mental and behavioral disorders due to psychoactive substance use; F2: Schizophrenia, schizotypal and delusional disorders; F3: Mood and affective disorders; F4: Neurotic, stress-related and somatoform disorders; F5: Behavioral syndromes associated with physiological disturbances and physical factors; F6: Disorders of adult personality and behavior; F7: Mental retardation; F8: Disorders of psychological development; F9: Behavioral and emotional disorders with onset usually occurring in childhood and adolescence

Overall, there was a significant association between the CAD and diagnostic group in adulthood, i.e. the distribution of adult diagnoses was dependent on the CAD (*χ*^2^ = 195.1, d.f. = 100, *p* < 0.001). The stability of the diagnostic category varied among ICD-10 diagnoses. Fairly high diagnostic stability was observed for the group F1 (substance abuse): 58.8 % of those previously diagnosed as F1 showed the same diagnosis as adults. Somewhat lower stability was observed in the groups F2 (schizophrenia, schizotypal and delusional disorders), F3 (mood/affective disorders) and F4 (neurotic, stress-related and somatoform disorders). In these categories, approximately 25 to 45 % of patients remained in the same group. A change in diagnosis was seen in the other groups. High transition probabilities were observed for transitions/shifts from a CAD of F9 (psychiatric disorders with onset in childhood or adolescence) to the adult diagnoses of F1 (substance abuse, 34.3 %) and F6 (personality disorder, 25.3 %), and also from a CAD of F4 (neurotic, stress-related and somatoform disorders) to an adult diagnosis of F1 (substance abuse, 24.1 %). Further details are given in Table 4.

## Discussion

### Sample

All minors who received a psychiatric inpatient diagnosis in Tyrol over a period of nearly two decades were included in our sample. The Department of Child and Adolescent Psychiatry in Innsbruck is the only facility with a public service mandate for the state. Children and adolescents in our inpatient sample showed characteristics comparable to population-based epidemiological studies in German-speaking countries [2], i.e. with anxiety and adjustment disorders as the largest diagnostic group, followed by childhood-specific diagnoses such as ADHD and conduct disorders. Young men were diagnosed more often with externalizing disorders, young women more often with internalizing disorders (statistcally significant findings). These patterns of gender distribution are also well known in population-based epidemiological studies [2, 16]. Together, this may be indicative of the representativeness of our sample and of the broader generalizability of our findings.

### Readmissions in adulthood as indicators of chronicity or reoccurrence

A striking finding from this investigation is that more than a quarter of our cohort of former CAP-IP used psychiatric inpatient health services again as adults, thus indicating chronicity or reoccurrence. This readmission rate is in line with previous findings from Germany and Scandinavia [26, 27]. It should be taken into account that in this study, we were only able to track cases within the federal state of Tyrol. Patients who had moved to other parts of Austria or to neighboring countries, such as Germany, Switzerland or Italy, were not included, but would certainly provide additional data. Furthermore, only inpatient cases (readmissions) were included; outpatient contacts with psychiatrists were not included but would likely also provide a large number of relevant data. Therefore, we may assume an even higher treatment rate. Data from social insurance carriers might shed further light on this issue.

Nevertheless, is it is possible to put the readmission rates of our cohort into a larger context by comparing them with admission rates of the general Austrian population. Figures from the Association of Austrian Social Security Institutions (published online, http://www.hauptverband.at) indicate that in 2007, only 0.8 % of all Austrians with social insurance were admitted to a psychiatric hospital. The patients of our cohort, by contrast, showed a much higher mean admission rate in adulthood of 4.5 % per year. This means that, compared with the general Austrian population, the odds of admission per year to a psychiatric hospital of our cohort of former CAP-IP were 5.6 times higher.

### Diagnostic transitions

The strongest continuity within our study was seen in the diagnostic group of substance use disorders (SUD), where nearly 60 % of juvenile SUD patients were found to receive this diagnosis again in adulthood. Thus, our results support previous findings that identify juvenile substance use problems as the main risk factor for a later SUD [23]. A diagnostic stability of nearly 45 % was also present for the group F2 (schizophrenia, schizotypal and delusional disorders), although it should be taken into account that this diagnostic group was small.

With regard to diagnostic change, the highest probability of transition was observed for a later SUD in adulthood. In line with previous findings [28–30], nearly 28 % of all juvenile patients developed an SUD, regardless of their CAD. Two diagnostic categories showed the highest transition rates to a later SUD: F9 (behavioral and emotional disorders with onset usually occurring in childhood and adolescence), with 34.1 %, and F4 (neurotic, stress-related and somatoform disorders), with 24.1 %. In sum, patients with juvenile ADHD, conduct disorder and anxiety disorders were frequently diagnosed with an SUD in adulthood.

Interestingly, a high transition probability was observed for a later inpatient diagnosis of a personality disorder: 17.2 % of former CAP-IP were diagnosed as suffering from a personality disorder in adulthood. The strongest predictor of this development was an ICD-10 CAD of F9 (behavioral and emotional disorders with onset usually occurring in childhood and adolescence): 25.3 % of the patients in this group were diagnosed with a personality disorder as inpatients in adulthood. This is in line with a Swedish study [31] in which childhood psychiatric morbidity unspecifically increased the risk for adult personality disorder symptoms. Both these findings and our findings call into question the usefulness of the restraint frequently exercised in diagnosing personality disorders in youth. Indeed, symptoms of personality pathology reach a first peak in adolescence and are a strong predictor of personality disorders associated with functional impairments and psychosocial difficulties in early adulthood [32]. Potentially, some of the patients in our cohort who developed a personality disorder in adulthood could have been diagnosed and treated more specifically in younger years.

Taken together, the findings point to three important patterns of diagnostic progression in our sample: first, young patients diagnosed with an SUD stayed in the same diagnostic category when they were treated as inpatients in adulthood; second, an SUD diagnosis in adulthood followed an ADHD, conduct disorder or an anxiety disorder in youth; and third, patients with childhood-specific psychiatric disorders in youth (e.g. ADHD or conduct disorders) were diagnosed with a personality disorder as inpatients in adulthood. However, as our study followed inpatient diagnoses between youth and adulthood and did not include information on outpatient treatment, we cannot rule out that patients from our sample may have shown additional patterns that are not indicated by our data.

### Socio-demographic and clinical variables

Multiple logistic regression showed no effect of gender, cumulative duration of inpatient stays or diagnostic group on readmission in adulthood. By contrast, large effects were found for age and number of admissions. Specifically, adolescent patients with multiple stays were at high risk for requiring psychiatric services again in adulthood. In our sample, the odds of chronicity were more than three times higher for those presenting as adolescents compared to childhood patients. This finding underscores the importance of early detection of mental health problems and of early intervention in preventing chronic illness. Moreover, adolescents with a large number of admissions to CAP seemed to be a high risk group and should be intensively monitored and, when necessary, treated.

### Strengths and limitations

This study is the first and only large-scale longitudinal study on the progression of mental health in children and adolescents in Austria to date. As government-supported prospective epidemiological surveys are not available in Austria, this study used pre-existing registry data to extract longitudinal trends via data matching. With this method, we were able to confirm established epidemiological knowledge with data from a cohort of Austrian youth. In addition to closing this gap, the study highlights the relevance of mental health problems of Austrian youth coming of age. Nevertheless, some limitations should be taken into account. First, we were only able to track cases within the federal state of Tyrol, and we did not have access to diagnostic data on outpatient cases, e.g. people treated as adults by psychiatrists in private practice. In other words, our survey only observed the development from CAP-IP cases to adult inpatient cases. We assume a much higher treatment rate but had no access to the relevant data at the time of our assessment. Second, our study is based on diagnostic data from specialized psychiatric hospitals, and these data were not gathered using standardized diagnostic instruments. We thus cannot exclude that these “best-estimate” diagnoses represent a source of potential bias. Third, our study focused on psychiatric main diagnoses derived from pre-existing clinical data, thus we could not take into account the full spectrum of potential comorbid diagnoses. While one of our main findings - a readmission-rate of 26 % - is not affected by comorbidity, some of the patterns of diagnostic progression described above could have shown different characteristics when taken comorbidity into account.

## Conclusions

The results of this study show that more than a quarter of all former patients of a specialized facility for child and adolescent psychiatry were also inpatients at psychiatric clinics as adults. The likelihood in our sample of former CAP-IP also requiring psychiatric inpatient treatment in adulthood is 5.6 times higher than for the general Austrian population. The findings point to two high-risk groups. First, older adolescents with several inpatient stays in short succession show a significantly higher risk of requiring inpatient treatment for a mental illness in adulthood. Second, 60 % of all CAP-IP with substance use problems show these same problems as inpatients in adulthood. Former child and adolescent psychiatry patients frequently develop addictive disorders, personality disorders, or adjustment and anxiety disorders in adulthood. The results thus suggest that mental illness in adulthood can, at least to some extent, be reconceptualized as a further development of mental illness beginning in childhood or adolescence.

## Abbreviations

CAD, child and/or adolescent diagnosis; CAP, child and adolescent psychiatry; CAP-IP, child and adolescent psychiatry inpatients; SUD, substance use disorder
